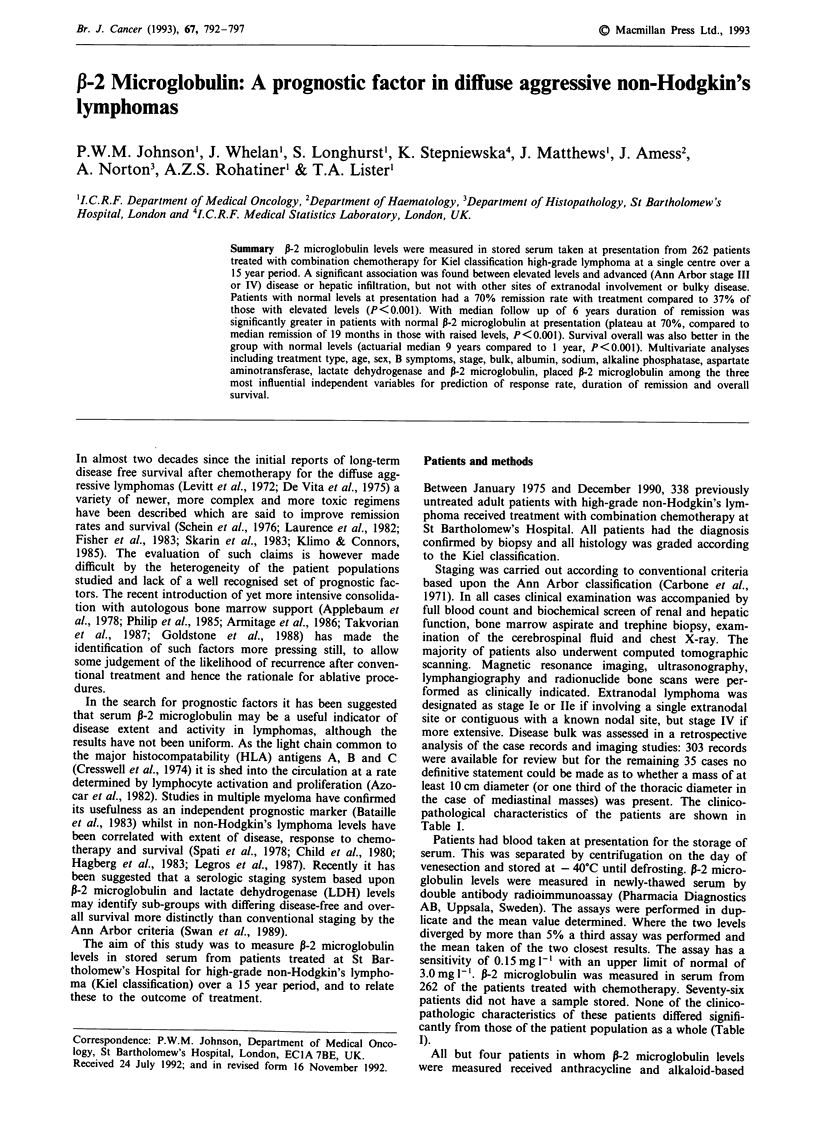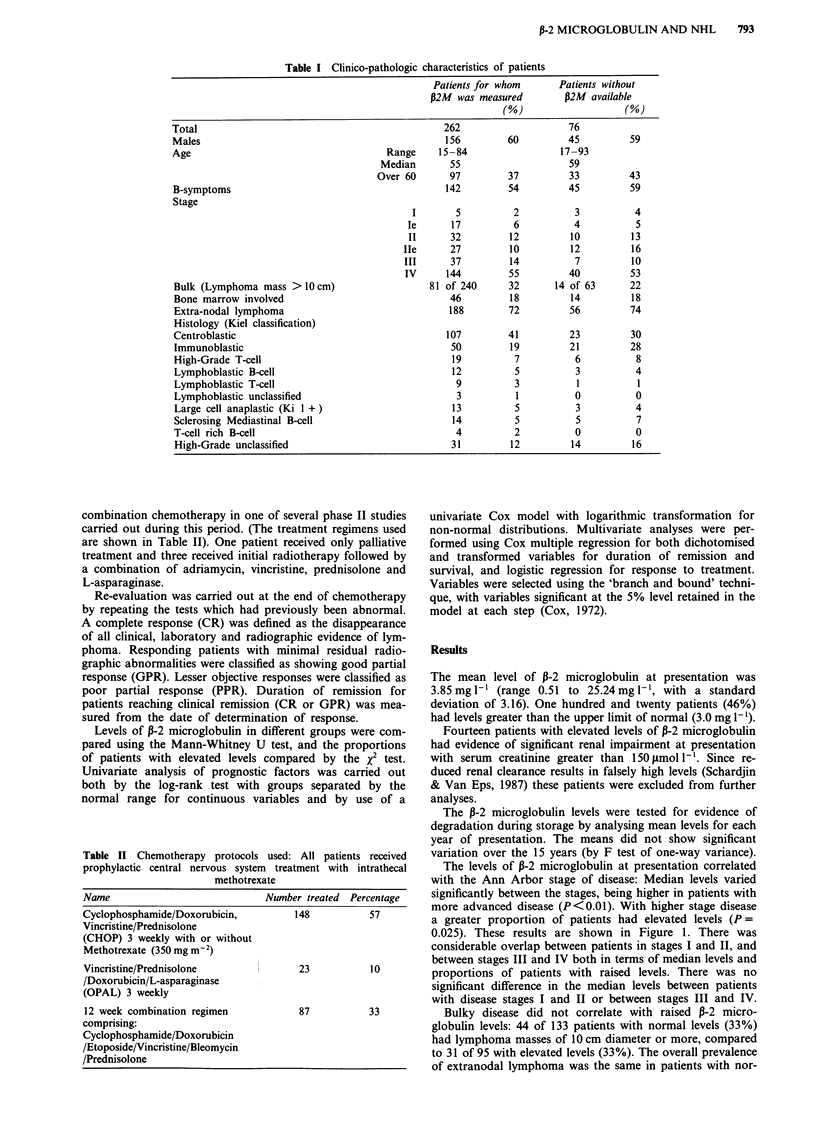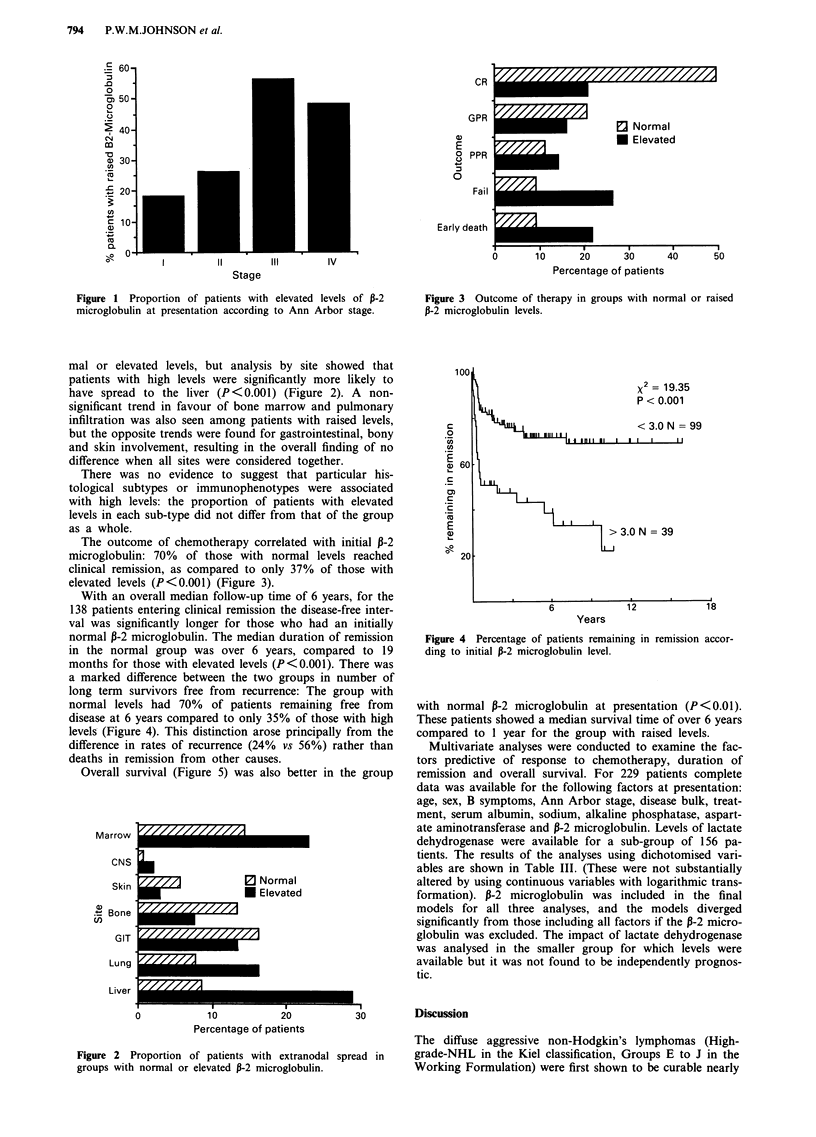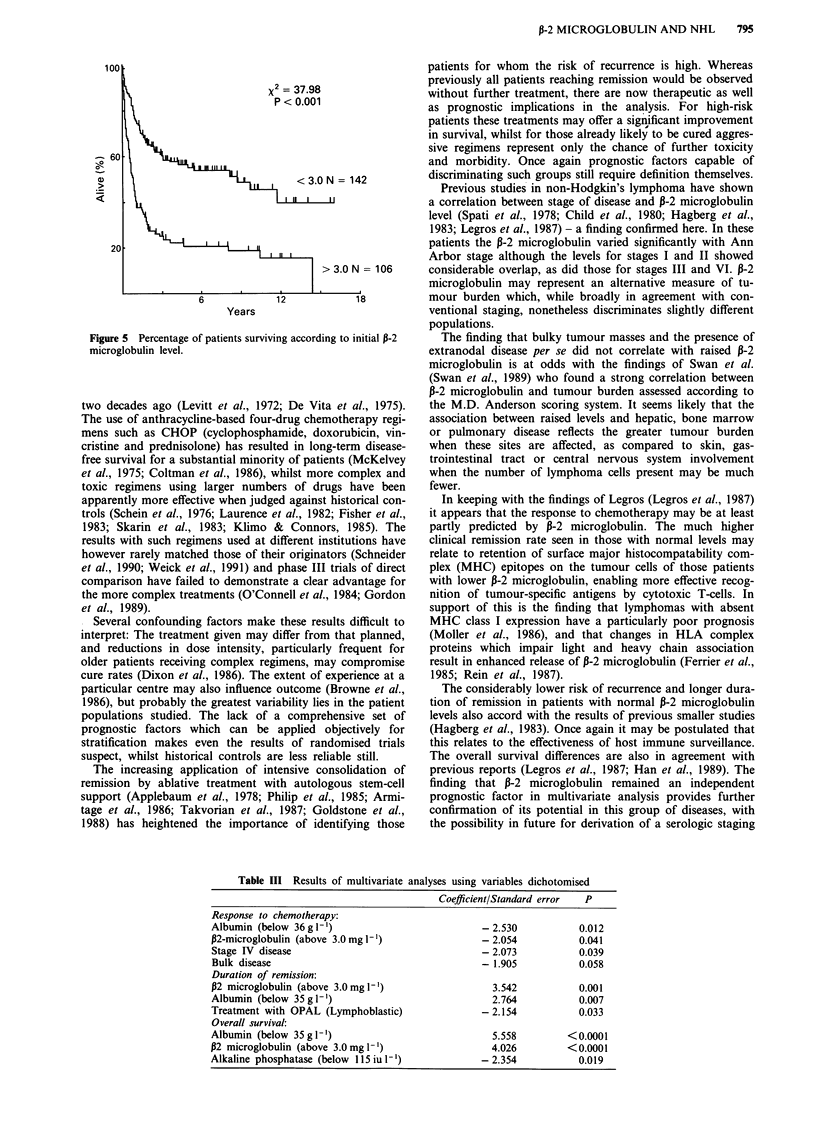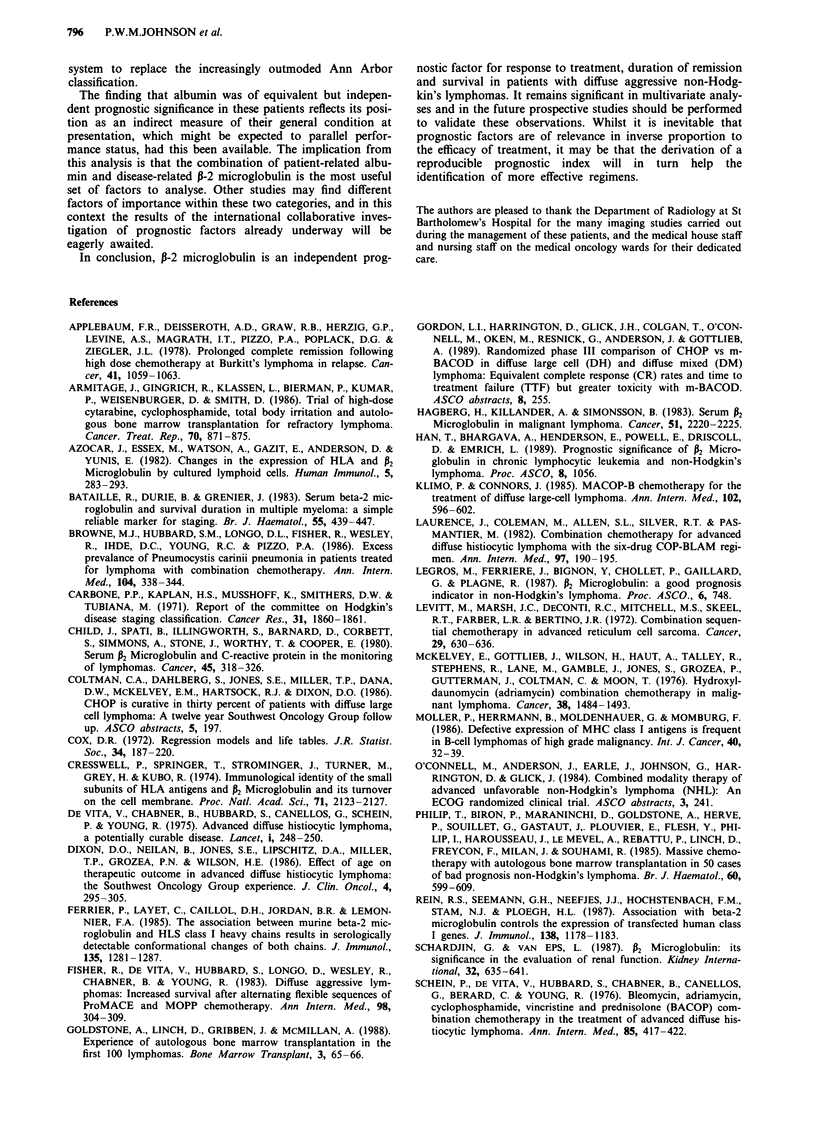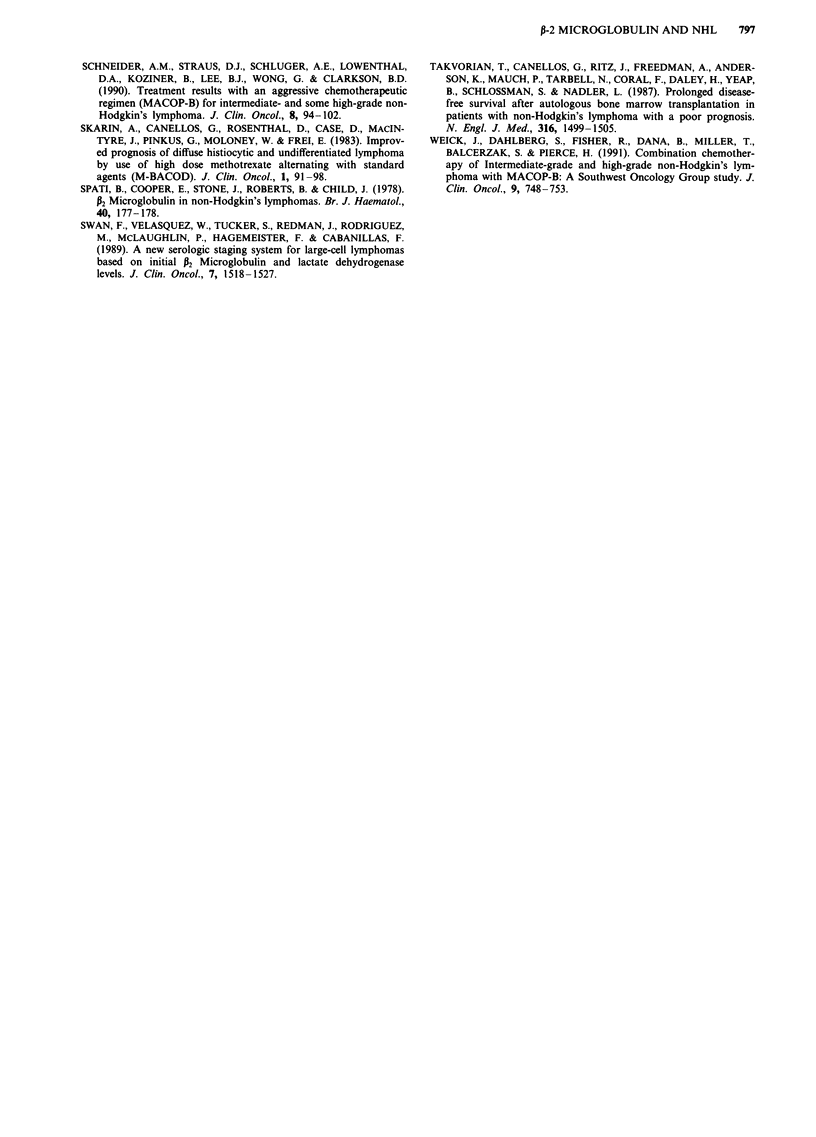# Beta-2 microglobulin: a prognostic factor in diffuse aggressive non-Hodgkin's lymphomas.

**DOI:** 10.1038/bjc.1993.144

**Published:** 1993-04

**Authors:** P. W. Johnson, J. Whelan, S. Longhurst, K. Stepniewska, J. Matthews, J. Amess, A. Norton, A. Z. Rohatiner, T. A. Lister

**Affiliations:** I.C.R.F. Department of Medical Oncology, St Bartholomew's Hospital, London, UK.

## Abstract

beta-2 microglobulin levels were measured in stored serum taken at presentation from 262 patients treated with combination chemotherapy for Kiel classification high-grade lymphoma at a single centre over a 15 year period. A significant association was found between elevated levels and advanced (Ann Arbor stage III or IV) disease or hepatic infiltration, but not with other sites of extranodal involvement or bulky disease. Patients with normal levels at presentation had a 70% remission rate with treatment compared to 37% of those with elevated levels (P < 0.001). With median follow up of 6 years duration of remission was significantly greater in patients with normal beta-2 microglobulin at presentation (plateau at 70%, compared to median remission of 19 months in those with raised levels, P < 0.001). Survival overall was also better in the group with normal levels (actuarial median 9 years compared to 1 year, P < 0.001). Multivariate analyses including treatment type, age, sex, B symptoms, stage, bulk, albumin, sodium, alkaline phosphatase, aspartate aminotransferase, lactate dehydrogenase and beta-2 microglobulin, placed beta-2 microglobulin among the three most influential independent variables for prediction of response rate, duration of remission and overall survival.


Br. J. Cancer (1993), 67, 792-797 © Macmillan Press Ltd., 1993

### β-2 Microglobulin: A prognostic factor in diffuse aggressive non-Hodgkin's lymphomas

P.W.M. Johnson[1], J. Whelan[1], S. Longhurst[1], K. Stepniewska[4], J. Matthews[1], J. Amess[2], A. Norton[3], A.Z.S. Rohatiner[1] & T.A. Lister[1]

[1]*I.C.R.F. Department of Medical Oncology,* [2]*Department of Haematology,* [3]*Department of Histopathology, St Bartholomew's Hospital, London and* [4]*I.C.R.F. Medical Statistics Laboratory, London, UK.*

**Summary** β-2 microglobulin levels were measured in stored serum taken at presentation from 262 patients treated with combination chemotherapy for Kiel classification high-grade lymphoma at a single centre over a 15 year period. A significant association was found between elevated levels and advanced (Ann Arbor stage III or IV) disease or hepatic infiltration, but not with other sites of extranodal involvement or bulky disease. Patients with normal levels at presentation had a 70% remission rate with treatment compared to 37% of those with elevated levels ($P<0.001$). With median follow up of 6 years duration of remission was significantly greater in patients with normal β-2 microglobulin at presentation (plateau at 70%, compared to median remission of 19 months in those with raised levels, $P<0.001$). Survival overall was also better in the group with normal levels (actuarial median 9 years compared to 1 year, $P<0.001$). Multivariate analyses including treatment type, age, sex, B symptoms, stage, bulk, albumin, sodium, alkaline phosphatase, aspartate aminotransferase, lactate dehydrogenase and β-2 microglobulin, placed β-2 microglobulin among the three most influential independent variables for prediction of response rate, duration of remission and overall survival.

In almost two decades since the initial reports of long-term disease free survival after chemotherapy for the diffuse aggressive lymphomas (Levitt *et al.*, 1972; De Vita *et al.*, 1975) a variety of newer, more complex and more toxic regimens have been described which are said to improve remission rates and survival (Schein *et al.*, 1976; Laurence *et al.*, 1982; Fisher *et al.*, 1983; Skarin *et al.*, 1983; Klimo & Connors, 1985). The evaluation of such claims is however made difficult by the heterogeneity of the patient populations studied and lack of a well recognised set of prognostic factors. The recent introduction of yet more intensive consolidation with autologous bone marrow support (Applebaum *et al.*, 1978; Philip *et al.*, 1985; Armitage *et al.*, 1986; Takvorian *et al.*, 1987; Goldstone *et al.*, 1988) has made the identification of such factors more pressing still, to allow some judgement of the likelihood of recurrence after conventional treatment and hence the rationale for ablative procedures.

In the search for prognostic factors it has been suggested that serum β-2 microglobulin may be a useful indicator of disease extent and activity in lymphomas, although the results have not been uniform. As the light chain common to the major histocompatability (HLA) antigens A, B and C (Cresswell *et al.*, 1974) it is shed into the circulation at a rate determined by lymphocyte activation and proliferation (Azocar *et al.*, 1982). Studies in multiple myeloma have confirmed its usefulness as an independent prognostic marker (Bataille *et al.*, 1983) whilst in non-Hodgkin's lymphoma levels have been correlated with extent of disease, response to chemotherapy and survival (Spati *et al.*, 1978; Child *et al.*, 1980; Hagberg *et al.*, 1983; Legros *et al.*, 1987). Recently it has been suggested that a serologic staging system based upon β-2 microglobulin and lactate dehydrogenase (LDH) levels may identify sub-groups with differing disease-free and overall survival more distinctly than conventional staging by the Ann Arbor criteria (Swan *et al.*, 1989).

The aim of this study was to measure β-2 microglobulin levels in stored serum from patients treated at St Bartholomew's Hospital for high-grade non-Hodgkin's lymphoma (Kiel classification) over a 15 year period, and to relate these to the outcome of treatment.

Correspondence: P.W.M. Johnson, Department of Medical Oncology, St Bartholomew's Hospital, London, EC1A 7BE, UK.
Received 24 July 1992; and in revised form 16 November 1992.

### Patients and methods

Between January 1975 and December 1990, 338 previously untreated adult patients with high-grade non-Hodgkin's lymphoma received treatment with combination chemotherapy at St Bartholomew's Hospital. All patients had the diagnosis confirmed by biopsy and all histology was graded according to the Kiel classification.

Staging was carried out according to conventional criteria based upon the Ann Arbor classification (Carbone *et al.*, 1971). In all cases clinical examination was accompanied by full blood count and biochemical screen of renal and hepatic function, bone marrow aspirate and trephine biopsy, examination of the cerebrospinal fluid and chest X-ray. The majority of patients also underwent computed tomographic scanning. Magnetic resonance imaging, ultrasonography, lymphangiography and radionuclide bone scans were performed as clinically indicated. Extranodal lymphoma was designated as stage Ie or IIe if involving a single extranodal site or contiguous with a known nodal site, but stage IV if more extensive. Disease bulk was assessed in a retrospective analysis of the case records and imaging studies: 303 records were available for review but for the remaining 35 cases no definitive statement could be made as to whether a mass of at least 10 cm diameter (or one third of the thoracic diameter in the case of mediastinal masses) was present. The clinicopathological characteristics of the patients are shown in Table I.

Patients had blood taken at presentation for the storage of serum. This was separated by centrifugation on the day of venesection and stored at − 40°C until defrosting. β-2 microglobulin levels were measured in newly-thawed serum by double antibody radioimmunoassay (Pharmacia Diagnostics AB, Uppsala, Sweden). The assays were performed in duplicate and the mean value determined. Where the two levels diverged by more than 5% a third assay was performed and the mean taken of the two closest results. The assay has a sensitivity of $0.15\,mg\,l^{-1}$ with an upper limit of normal of $3.0\,mg\,l^{-1}$. β-2 microglobulin was measured in serum from 262 of the patients treated with chemotherapy. Seventy-six patients did not have a sample stored. None of the clinicopathologic characteristics of these patients differed significantly from those of the patient population as a whole (Table I).

All but four patients in whom β-2 microglobulin levels were measured received anthracycline and alkaloid-based

**Table I** Clinico-pathologic characteristics of patients

| | | *Patients for whom β2M was measured* | *(%)* | *Patients without β2M available* | *(%)* |
|---|---|---|---|---|---|
| Total | | 262 | | 76 | |
| Males | | 156 | 60 | 45 | 59 |
| Age | Range | 15–84 | | 17–93 | |
| | Median | 55 | | 59 | |
| | Over 60 | 97 | 37 | 33 | 43 |
| B-symptoms | | 142 | 54 | 45 | 59 |
| Stage | | | | | |
| | I | 5 | 2 | 3 | 4 |
| | Ie | 17 | 6 | 4 | 5 |
| | II | 32 | 12 | 10 | 13 |
| | IIe | 27 | 10 | 12 | 16 |
| | III | 37 | 14 | 7 | 10 |
| | IV | 144 | 55 | 40 | 53 |
| Bulk (Lymphoma mass >10 cm) | | 81 of 240 | 32 | 14 of 63 | 22 |
| Bone marrow involved | | 46 | 18 | 14 | 18 |
| Extra-nodal lymphoma | | 188 | 72 | 56 | 74 |
| Histology (Kiel classification) | | | | | |
| Centroblastic | | 107 | 41 | 23 | 30 |
| Immunoblastic | | 50 | 19 | 21 | 28 |
| High-Grade T-cell | | 19 | 7 | 6 | 8 |
| Lymphoblastic B-cell | | 12 | 5 | 3 | 4 |
| Lymphoblastic T-cell | | 9 | 3 | 1 | 1 |
| Lymphoblastic unclassified | | 3 | 1 | 0 | 0 |
| Large cell anaplastic (Ki 1+) | | 13 | 5 | 3 | 4 |
| Sclerosing Mediastinal B-cell | | 14 | 5 | 5 | 7 |
| T-cell rich B-cell | | 4 | 2 | 0 | 0 |
| High-Grade unclassified | | 31 | 12 | 14 | 16 |

combination chemotherapy in one of several phase II studies carried out during this period. (The treatment regimens used are shown in Table II). One patient received only palliative treatment and three received initial radiotherapy followed by a combination of adriamycin, vincristine, prednisolone and L-asparaginase.

Re-evaluation was carried out at the end of chemotherapy by repeating the tests which had previously been abnormal. A complete response (CR) was defined as the disappearance of all clinical, laboratory and radiographic evidence of lymphoma. Responding patients with minimal residual radiographic abnormalities were classified as showing good partial response (GPR). Lesser objective responses were classified as poor partial response (PPR). Duration of remission for patients reaching clinical remission (CR or GPR) was measured from the date of determination of response.

Levels of β-2 microglobulin in different groups were compared using the Mann-Whitney U test, and the proportions of patients with elevated levels compared by the $\chi^2$ test. Univariate analysis of prognostic factors was carried out both by the log-rank test with groups separated by the normal range for continuous variables and by use of a univariate Cox model with logarithmic transformation for non-normal distributions. Multivariate analyses were performed using Cox multiple regression for both dichotomised and transformed variables for duration of remission and survival, and logistic regression for response to treatment. Variables were selected using the 'branch and bound' technique, with variables significant at the 5% level retained in the model at each step (Cox, 1972).

**Table II** Chemotherapy protocols used: All patients received prophylactic central nervous system treatment with intrathecal methotrexate

| *Name* | *Number treated* | *Percentage* |
|---|---|---|
| Cyclophosphamide/Doxorubicin, Vincristine/Prednisolone (CHOP) 3 weekly with or without Methotrexate (350 mg m$^{-2}$) | 148 | 57 |
| Vincristine/Prednisolone /Doxorubicin/L-asparaginase (OPAL) 3 weekly | 23 | 10 |
| 12 week combination regimen comprising: Cyclophosphamide/Doxorubicin /Etoposide/Vincristine/Bleomycin /Prednisolone | 87 | 33 |

### Results

The mean level of β-2 microglobulin at presentation was 3.85 mg l$^{-1}$ (range 0.51 to 25.24 mg l$^{-1}$, with a standard deviation of 3.16). One hundred and twenty patients (46%) had levels greater than the upper limit of normal (3.0 mg l$^{-1}$).

Fourteen patients with elevated levels of β-2 microglobulin had evidence of significant renal impairment at presentation with serum creatinine greater than 150 μmol l$^{-1}$. Since reduced renal clearance results in falsely high levels (Schardjin & Van Eps, 1987) these patients were excluded from further analyses.

The β-2 microglobulin levels were tested for evidence of degradation during storage by analysing mean levels for each year of presentation. The means did not show significant variation over the 15 years (by F test of one-way variance).

The levels of β-2 microglobulin at presentation correlated with the Ann Arbor stage of disease: Median levels varied significantly between the stages, being higher in patients with more advanced disease ($P<0.01$). With higher stage disease a greater proportion of patients had elevated levels ($P=0.025$). These results are shown in Figure 1. There was considerable overlap between patients in stages I and II, and between stages III and IV both in terms of median levels and proportions of patients with raised levels. There was no significant difference in the median levels between patients with disease stages I and II or between stages III and IV.

Bulky disease did not correlate with raised β-2 microglobulin levels: 44 of 133 patients with normal levels (33%) had lymphoma masses of 10 cm diameter or more, compared to 31 of 95 with elevated levels (33%). The overall prevalence of extranodal lymphoma was the same in patients with nor-

**Figure 1** Proportion of patients with elevated levels of β-2 microglobulin at presentation according to Ann Arbor stage.

**Figure 3** Outcome of therapy in groups with normal or raised β-2 microglobulin levels.

mal or elevated levels, but analysis by site showed that patients with high levels were significantly more likely to have spread to the liver ($P<0.001$) (Figure 2). A non-significant trend in favour of bone marrow and pulmonary infiltration was also seen among patients with raised levels, but the opposite trends were found for gastrointestinal, bony and skin involvement, resulting in the overall finding of no difference when all sites were considered together.

There was no evidence to suggest that particular histological subtypes or immunophenotypes were associated with high levels: the proportion of patients with elevated levels in each sub-type did not differ from that of the group as a whole.

The outcome of chemotherapy correlated with initial β-2 microglobulin: 70% of those with normal levels reached clinical remission, as compared to only 37% of those with elevated levels ($P<0.001$) (Figure 3).

With an overall median follow-up time of 6 years, for the 138 patients entering clinical remission the disease-free interval was significantly longer for those who had an initially normal β-2 microglobulin. The median duration of remission in the normal group was over 6 years, compared to 19 months for those with elevated levels ($P<0.001$). There was a marked difference between the two groups in number of long term survivors free from recurrence: The group with normal levels had 70% of patients remaining free from disease at 6 years compared to only 35% of those with high levels (Figure 4). This distinction arose principally from the difference in rates of recurrence (24% *vs* 56%) rather than deaths in remission from other causes.

Overall survival (Figure 5) was also better in the group

**Figure 2** Proportion of patients with extranodal spread in groups with normal or elevated β-2 microglobulin.

**Figure 4** Percentage of patients remaining in remission according to initial β-2 microglobulin level.

with normal β-2 microglobulin at presentation ($P<0.01$). These patients showed a median survival time of over 6 years compared to 1 year for the group with raised levels.

Multivariate analyses were conducted to examine the factors predictive of response to chemotherapy, duration of remission and overall survival. For 229 patients complete data was available for the following factors at presentation: age, sex, B symptoms, Ann Arbor stage, disease bulk, treatment, serum albumin, sodium, alkaline phosphatase, aspartate aminotransferase and β-2 microglobulin. Levels of lactate dehydrogenase were available for a sub-group of 156 patients. The results of the analyses using dichotomised variables are shown in Table III. (These were not substantially altered by using continuous variables with logarithmic transformation). β-2 microglobulin was included in the final models for all three analyses, and the models diverged significantly from those including all factors if the β-2 microglobulin was excluded. The impact of lactate dehydrogenase was analysed in the smaller group for which levels were available but it was not found to be independently prognostic.

### Discussion

The diffuse aggressive non-Hodgkin's lymphomas (High-grade-NHL in the Kiel classification, Groups E to J in the Working Formulation) were first shown to be curable nearly

**Figure 5** Percentage of patients surviving according to initial β-2 microglobulin level.

two decades ago (Levitt *et al.*, 1972; De Vita *et al.*, 1975). The use of anthracycline-based four-drug chemotherapy regimens such as CHOP (cyclophosphamide, doxorubicin, vincristine and prednisolone) has resulted in long-term disease-free survival for a substantial minority of patients (McKelvey *et al.*, 1975; Coltman *et al.*, 1986), whilst more complex and toxic regimens using larger numbers of drugs have been apparently more effective when judged against historical controls (Schein *et al.*, 1976; Laurence *et al.*, 1982; Fisher *et al.*, 1983; Skarin *et al.*, 1983; Klimo & Connors, 1985). The results with such regimens used at different institutions have however rarely matched those of their originators (Schneider *et al.*, 1990; Weick *et al.*, 1991) and phase III trials of direct comparison have failed to demonstrate a clear advantage for the more complex treatments (O'Connell *et al.*, 1984; Gordon *et al.*, 1989).

Several confounding factors make these results difficult to interpret: The treatment given may differ from that planned, and reductions in dose intensity, particularly frequent for older patients receiving complex regimens, may compromise cure rates (Dixon *et al.*, 1986). The extent of experience at a particular centre may also influence outcome (Browne *et al.*, 1986), but probably the greatest variability lies in the patient populations studied. The lack of a comprehensive set of prognostic factors which can be applied objectively for stratification makes even the results of randomised trials suspect, whilst historical controls are less reliable still.

The increasing application of intensive consolidation of remission by ablative treatment with autologous stem-cell support (Applebaum *et al.*, 1978; Philip *et al.*, 1985; Armitage *et al.*, 1986; Takvorian *et al.*, 1987; Goldstone *et al.*, 1988) has heightened the importance of identifying those patients for whom the risk of recurrence is high. Whereas previously all patients reaching remission would be observed without further treatment, there are now therapeutic as well as prognostic implications in the analysis. For high-risk patients these treatments may offer a significant improvement in survival, whilst for those already likely to be cured aggressive regimens represent only the chance of further toxicity and morbidity. Once again prognostic factors capable of discriminating such groups still require definition themselves.

Previous studies in non-Hodgkin's lymphoma have shown a correlation between stage of disease and β-2 microglobulin level (Spati *et al.*, 1978; Child *et al.*, 1980; Hagberg *et al.*, 1983; Legros *et al.*, 1987) – a finding confirmed here. In these patients the β-2 microglobulin varied significantly with Ann Arbor stage although the levels for stages I and II showed considerable overlap, as did those for stages III and VI. β-2 microglobulin may represent an alternative measure of tumour burden which, while broadly in agreement with conventional staging, nonetheless discriminates slightly different populations.

The finding that bulky tumour masses and the presence of extranodal disease *per se* did not correlate with raised β-2 microglobulin is at odds with the findings of Swan *et al.* (Swan *et al.*, 1989) who found a strong correlation between β-2 microglobulin and tumour burden assessed according to the M.D. Anderson scoring system. It seems likely that the association between raised levels and hepatic, bone marrow or pulmonary disease reflects the greater tumour burden when these sites are affected, as compared to skin, gastrointestinal tract or central nervous system involvement when the number of lymphoma cells present may be much fewer.

In keeping with the findings of Legros (Legros *et al.*, 1987) it appears that the response to chemotherapy may be at least partly predicted by β-2 microglobulin. The much higher clinical remission rate seen in those with normal levels may relate to retention of surface major histocompatability complex (MHC) epitopes on the tumour cells of those patients with lower β-2 microglobulin, enabling more effective recognition of tumour-specific antigens by cytotoxic T-cells. In support of this is the finding that lymphomas with absent MHC class I expression have a particularly poor prognosis (Moller *et al.*, 1986), and that changes in HLA complex proteins which impair light and heavy chain association result in enhanced release of β-2 microglobulin (Ferrier *et al.*, 1985; Rein *et al.*, 1987).

The considerably lower risk of recurrence and longer duration of remission in patients with normal β-2 microglobulin levels also accord with the results of previous smaller studies (Hagberg *et al.*, 1983). Once again it may be postulated that this relates to the effectiveness of host immune surveillance. The overall survival differences are also in agreement with previous reports (Legros *et al.*, 1987; Han *et al.*, 1989). The finding that β-2 microglobulin remained an independent prognostic factor in multivariate analysis provides further confirmation of its potential in this group of diseases, with the possibility in future for derivation of a serologic staging

**Table III** Results of multivariate analyses using variables dichotomised

| | *Coefficient/Standard error* | *P* |
|---|---|---|
| *Response to chemotherapy*: | | |
| Albumin (below 36 g l$^{-1}$) | – 2.530 | 0.012 |
| β2-microglobulin (above 3.0 mg l$^{-1}$) | – 2.054 | 0.041 |
| Stage IV disease | – 2.073 | 0.039 |
| Bulk disease | – 1.905 | 0.058 |
| *Duration of remission*: | | |
| β2 microglobulin (above 3.0 mg l$^{-1}$) | 3.542 | 0.001 |
| Albumin (below 35 g l$^{-1}$) | 2.764 | 0.007 |
| Treatment with OPAL (Lymphoblastic) | – 2.154 | 0.033 |
| *Overall survival*: | | |
| Albumin (below 35 g l$^{-1}$) | 5.558 | <0.0001 |
| β2 microglobulin (above 3.0 mg l$^{-1}$) | 4.026 | <0.0001 |
| Alkaline phosphatase (below 115 iu l$^{-1}$) | – 2.354 | 0.019 |

system to replace the increasingly outmoded Ann Arbor classification.

The finding that albumin was of equivalent but independent prognostic significance in these patients reflects its position as an indirect measure of their general condition at presentation, which might be expected to parallel performance status, had this been available. The implication from this analysis is that the combination of patient-related albumin and disease-related $\beta$-2 microglobulin is the most useful set of factors to analyse. Other studies may find different factors of importance within these two categories, and in this context the results of the international collaborative investigation of prognostic factors already underway will be eagerly awaited.

In conclusion, $\beta$-2 microglobulin is an independent prognostic factor for response to treatment, duration of remission and survival in patients with diffuse aggressive non-Hodgkin's lymphomas. It remains significant in multivariate analyses and in the future prospective studies should be performed to validate these observations. Whilst it is inevitable that prognostic factors are of relevance in inverse proportion to the efficacy of treatment, it may be that the derivation of a reproducible prognostic index will in turn help the identification of more effective regimens.

The authors are pleased to thank the Department of Radiology at St Bartholomew's Hospital for the many imaging studies carried out during the management of these patients, and the medical house staff and nursing staff on the medical oncology wards for their dedicated care.

### References

APPLEBAUM, F.R., DEISSEROTH, A.D., GRAW, R.B., HERZIG, G.P., LEVINE, A.S., MAGRATH, I.T., PIZZO, P.A., POPLACK, D.G. & ZIEGLER, J.L. (1978). Prolonged complete remission following high dose chemotherapy at Burkitt's lymphoma in relapse. *Cancer*, **41**, 1059–1063.

ARMITAGE, J., GINGRICH, R., KLASSEN, L., BIERMAN, P., KUMAR, P., WEISENBURGER, D. & SMITH, D. (1986). Trial of high-dose cytarabine, cyclophosphamide, total body irritation and autologous bone marrow transplantation for refractory lymphoma. *Cancer. Treat. Rep.*, **70**, 871–875.

AZOCAR, J., ESSEX, M., WATSON, A., GAZIT, E., ANDERSON, D. & YUNIS, E. (1982). Changes in the expression of HLA and $\beta_2$ Microglobulin by cultured lymphoid cells. *Human Immunol.*, **5**, 283–293.

BATAILLE, R., DURIE, B. & GRENIER, J. (1983). Serum beta-2 microglobulin and survival duration in multiple myeloma: a simple reliable marker for staging. *Br. J. Haematol.*, **55**, 439–447.

BROWNE, M.J., HUBBARD, S.M., LONGO, D.L., FISHER, R., WESLEY, R., IHDE, D.C., YOUNG, R.C. & PIZZO, P.A. (1986). Excess prevalance of Pneumocystis carinii pneumonia in patients treated for lymphoma with combination chemotherapy. *Ann. Intern. Med.*, **104**, 338–344.

CARBONE, P.P., KAPLAN, H.S., MUSSHOFF, K., SMITHERS, D.W. & TUBIANA, M. (1971). Report of the committee on Hodgkin's disease staging classification. *Cancer Res.*, **31**, 1860–1861.

CHILD, J., SPATI, B., ILLINGWORTH, S., BARNARD, D., CORBETT, S., SIMMONS, A., STONE, J., WORTHY, T. & COOPER, E. (1980). Serum $\beta_2$ Microglobulin and C-reactive protein in the monitoring of lymphomas. *Cancer*, **45**, 318–326.

COLTMAN, C.A., DAHLBERG, S., JONES, S.E., MILLER, T.P., DANA, D.W., MCKELVEY, E.M., HARTSOCK, R.J. & DIXON, D.O. (1986). CHOP is curative in thirty percent of patients with diffuse large cell lymphoma: A twelve year Southwest Oncology Group follow up. *ASCO abstracts*, **5**, 197.

COX, D.R. (1972). Regression models and life tables. *J.R. Statist. Soc.*, **34**, 187–220.

CRESSWELL, P., SPRINGER, T., STROMINGER, J., TURNER, M., GREY, H. & KUBO, R. (1974). Immunological identity of the small subunits of HLA antigens and $\beta_2$ Microglobulin and its turnover on the cell membrane. *Proc. Natl. Acad. Sci.*, **71**, 2123–2127.

DE VITA, V., CHABNER, B., HUBBARD, S., CANELLOS, G., SCHEIN, P. & YOUNG, R. (1975). Advanced diffuse histiocytic lymphoma, a potentially curable disease. *Lancet*, **i**, 248–250.

DIXON, D.O., NEILAN, B., JONES, S.E., LIPSCHITZ, D.A., MILLER, T.P., GROZEA, P.N. & WILSON, H.E. (1986). Effect of age on therapeutic outcome in advanced diffuse histiocytic lymphoma: the Southwest Oncology Group experience. *J. Clin. Oncol.*, **4**, 295–305.

FERRIER, P., LAYET, C., CAILLOL, D.H., JORDAN, B.R. & LEMONNIER, F.A. (1985). The association between murine beta-2 microglobulin and HLS class I heavy chains results in serologically detectable conformational changes of both chains. *J. Immunol.*, **135**, 1281–1287.

FISHER, R., DE VITA, V., HUBBARD, S., LONGO, D., WESLEY, R., CHABNER, B. & YOUNG, R. (1983). Diffuse aggressive lymphomas: Increased survival after alternating flexible sequences of ProMACE and MOPP chemotherapy. *Ann Intern. Med.*, **98**, 304–309.

GOLDSTONE, A., LINCH, D., GRIBBEN, J. & MCMILLAN, A. (1988). Experience of autologous bone marrow transplantation in the first 100 lymphomas. *Bone Marrow Transplant*, **3**, 65–66.

GORDON, L.I., HARRINGTON, D., GLICK, J.H., COLGAN, T., O'CONNELL, M., OKEN, M., RESNICK, G., ANDERSON, J. & GOTTLIEB, A. (1989). Randomized phase III comparison of CHOP vs m-BACOD in diffuse large cell (DH) and diffuse mixed (DM) lymphoma: Equivalent complete response (CR) rates and time to treatment failure (TTF) but greater toxicity with m-BACOD. *ASCO abstracts*, **8**, 255.

HAGBERG, H., KILLANDER, A. & SIMONSSON, B. (1983). Serum $\beta_2$ Microglobulin in malignant lymphoma. *Cancer*, **51**, 2220–2225.

HAN, T., BHARGAVA, A., HENDERSON, E., POWELL, E., DRISCOLL, D. & EMRICH, L. (1989). Prognostic significance of $\beta_2$ Microglobulin in chronic lymphocytic leukemia and non-Hodgkin's lymphoma. *Proc. ASCO*, **8**, 1056.

KLIMO, P. & CONNORS, J. (1985). MACOP-B chemotherapy for the treatment of diffuse large-cell lymphoma. *Ann. Intern. Med.*, **102**, 596–602.

LAURENCE, J., COLEMAN, M., ALLEN, S.L., SILVER, R.T. & PASMANTIER, M. (1982). Combination chemotherapy for advanced diffuse histiocytic lymphoma with the six-drug COP-BLAM regimen. *Ann. Intern. Med.*, **97**, 190–195.

LEGROS, M., FERRIERE, J., BIGNON, Y, CHOLLET, P., GAILLARD, G. & PLAGNE, R. (1987). $\beta_2$ Microglobulin: a good prognosis indicator in non-Hodgkin's lymphoma. *Proc. ASCO.*, **6**, 748.

LEVITT, M., MARSH, J.C., DECONTI, R.C., MITCHELL, M.S., SKEEL, R.T., FARBER, L.R. & BERTINO, J.R. (1972). Combination sequential chemotherapy in advanced reticulum cell sarcoma. *Cancer*, **29**, 630–636.

MCKELVEY, E., GOTTLIEB, J., WILSON, H., HAUT, A., TALLEY, R., STEPHENS, R., LANE, M., GAMBLE, J., JONES, S., GROZEA, P., GUTTERMAN, J., COLTMAN, C. & MOON, T. (1976). Hydroxyldaunomycin (adriamycin) combination chemotherapy in malignant lymphoma. *Cancer*, **38**, 1484–1493.

MOLLER, P., HERRMANN, B., MOLDENHAUER, G. & MOMBURG, F. (1986). Defective expression of MHC class I antigens is frequent in B-cell lymphomas of high grade malignancy. *Int. J. Cancer*, **40**, 32–39.

O'CONNELL, M., ANDERSON, J., EARLE, J., JOHNSON, G., HARRINGTON, D. & GLICK, J. (1984). Combined modality therapy of advanced unfavorable non-Hodgkin's lymphoma (NHL): An ECOG randomized clinical trial. *ASCO abstracts*, **3**, 241.

PHILIP, T., BIRON, P., MARANINCHI, D., GOLDSTONE, A., HERVE, P., SOUILLET, G., GASTAUT, J,. PLOUVIER, E., FLESH, Y., PHILIP, I., HAROUSSEAU, J., LE MEVEL, A., REBATTU, P., LINCH, D., FREYCON, F., MILAN, J. & SOUHAMI, R. (1985). Massive chemotherapy with autologous bone marrow transplantation in 50 cases of bad prognosis non-Hodgkin's lymphoma. *Br. J. Haematol.*, **60**, 599–609.

REIN, R.S., SEEMANN, G.H., NEEFJES, J.J., HOCHSTENBACH, F.M., STAM, N.J. & PLOEGH, H.L. (1987). Association with beta-2 microglobulin controls the expression of transfected human class I genes. *J. Immunol.*, **138**, 1178–1183.

SCHARDJIN, G. & VAN EPS, L. (1987). $\beta_2$ Microglobulin: its significance in the evaluation of renal function. *Kidney International*, **32**, 635–641.

SCHEIN, P., DE VITA, V., HUBBARD, S., CHABNER, B., CANELLOS, G., BERARD, C. & YOUNG, R. (1976). Bleomycin, adriamycin, cyclophosphamide, vincristine and prednisolone (BACOP) combination chemotherapy in the treatment of advanced diffuse histiocytic lymphoma. *Ann. Intern. Med.*, **85**, 417–422.

SCHNEIDER, A.M., STRAUS, D.J., SCHLUGER, A.E., LOWENTHAL, D.A., KOZINER, B., LEE, B.J., WONG, G. & CLARKSON, B.D. (1990). Treatment results with an aggressive chemotherapeutic regimen (MACOP-B) for intermediate- and some high-grade non-Hodgkin's lymphoma. *J. Clin. Oncol.*, **8**, 94–102.

SKARIN, A., CANELLOS, G., ROSENTHAL, D., CASE, D., MACINTYRE, J., PINKUS, G., MOLONEY, W. & FREI, E. (1983). Improved prognosis of diffuse histiocytic and undifferentiated lymphoma by use of high dose methotrexate alternating with standard agents (M-BACOD). *J. Clin. Oncol.*, **1**, 91–98.

SPATI, B., COOPER, E., STONE, J., ROBERTS, B. & CHILD, J. (1978). $\beta_2$ Microglobulin in non-Hodgkin's lymphomas. *Br. J. Haematol.*, **40**, 177–178.

SWAN, F., VELASQUEZ, W., TUCKER, S., REDMAN, J., RODRIGUEZ, M., MCLAUGHLIN, P., HAGEMEISTER, F. & CABANILLAS, F. (1989). A new serologic staging system for large-cell lymphomas based on initial $\beta_2$ Microglobulin and lactate dehydrogenase levels. *J. Clin. Oncol.*, **7**, 1518–1527.

TAKVORIAN, T., CANELLOS, G., RITZ, J., FREEDMAN, A., ANDERSON, K., MAUCH, P., TARBELL, N., CORAL, F., DALEY, H., YEAP, B., SCHLOSSMAN, S. & NADLER, L. (1987). Prolonged disease-free survival after autologous bone marrow transplantation in patients with non-Hodgkin's lymphoma with a poor prognosis. *N. Engl. J. Med.*, **316**, 1499–1505.

WEICK, J., DAHLBERG, S., FISHER, R., DANA, B., MILLER, T., BALCERZAK, S. & PIERCE, H. (1991). Combination chemotherapy of Intermediate-grade and high-grade non-Hodgkin's lymphoma with MACOP-B: A Southwest Oncology Group study. *J. Clin. Oncol.*, **9**, 748–753.